# Non-linear association between low-density lipoprotein cholesterol and risk of prediabetes: a retrospective cohort study based on Chinese adults

**DOI:** 10.3389/fendo.2025.1591893

**Published:** 2025-06-30

**Authors:** Long He, Yan Zhang, Wei Sha

**Affiliations:** ^1^ Organ Transplant Center, General Hospital of Northern Theater Command, Shenyang, China; ^2^ Department of Urology, Xuyi Clinical College, Medical College of Yangzhou University, Huaian, Jiangsu, China

**Keywords:** low-density lipoprotein cholesterol, prediabetes, cohort study, nonlinearly, prevention

## Abstract

**Background:**

The pathogenesis of prediabetes remains complex, particularly regarding the interactions between lipid metabolism disorders and glucose metabolism abnormalities, which warrant in-depth exploration. Low-density lipoprotein cholesterol (LDL-C) is an important risk factor for atherosclerosis and cardiovascular disease. However, the relationship between LDL-C and prediabetes has been less extensively studied. Therefore, we conducted a retrospective cohort study to investigate this association.

**Methods:**

This secondary retrospective cohort study utilized data from 100,608 Chinese adults. Cox proportional hazards regression models were used to examine the relationship between LDL-C and prediabetes risk. Restricted cubic spline regression and smooth curve fitting were used to explore the non-linear relationship between LDL-C and prediabetes. A two-piecewise Cox proportional hazards regression model identified inflection points. In addition, a series of subgroup and sensitivity analyses were performed to confirm the robustness of our results.

**Results:**

After adjusting for confounding covariates, LDL-C was positively associated with prediabetes (HR: 1.49, 95% CI: 1.40–1.58, p < 0.0001). The two-piecewise Cox model identified an inflection point of 2.19 for LDL-C (p < 0.001 for log-likelihood ratio test). When LDL-C ≤ 2.19, LDL-C was positively associated with the risk of prediabetes (HR: 2.02, 95% CI: 1.71–2.36, p < 0.0001). In contrast, when LDL-C > 2.19, LDL-C was associated with a lower risk of prediabetes (HR: 1.49, 95% CI: 1.39–1.59, p < 0.0001). Sensitivity and subgroup analyses confirmed the stability and consistency of this positive association in the general population.

**Conclusion:**

This study reveals a non-linear positive association between LDL-C levels and prediabetes risk in Chinese adults after adjusting for confounders. The dynamic monitoring of LDL-C levels may help identify individuals at high risk for prediabetes. Timely dietary and lifestyle modifications could potentially reduce the risk of prediabetes. These findings offer new insights for prediabetes prevention and treatment.

## Background

Prediabetes, also known as impaired glucose regulation, is a metabolic state characterized by blood glucose levels intermediate between normal and the diagnostic threshold for diabetes mellitus. It represents a critical early stage in the development of diabetes (1). The World Health Organization (WHO) and the American Diabetes Association (ADA) define the diagnostic criteria for prediabetes as impaired fasting glucose (IFG; 5.6–6.9 mmol/L) and/or abnormal glucose tolerance (IGT, 2-hour postprandial glucose 7.8–11.0 mmol/L) (2). The global prevalence of prediabetes is rising, with the International Diabetes Federation projecting 548 million affected adults by 2045 (3). Approximately 10%–30% of people with prediabetes will progress to type 2 diabetes within 5 years (4). Furthermore, prediabetes significantly increases the risk of cardiovascular disease and microangiopathy (5). In recent years, important advances have been made in intervention research targeting prediabetes. Lifestyle modifications (e.g., smoking cessation, dietary optimization, and physical activity) have been shown to significantly reduce the risk of progression from prediabetes to diabetes (6). A randomized controlled trial of patients with prediabetes showed a 50% reduction in 3-year diabetes incidence in patients who combined lifestyle and pharmacological interventions (7). In addition, the discovery of several novel biomarkers (e.g., plasma atherosclerotic index and residual cholesterol) has further optimized the individualized risk assessment and management of prediabetes (8, 9).

The pathogenesis of prediabetes remains complex, particularly concerning the interaction between lipid metabolism disorders and glucose metabolism abnormalities, which requires further investigation. Low-density lipoprotein cholesterol (LDL-C) is a major risk factor for atherosclerosis and cardiovascular disease, and recent studies have suggested a potential association with prediabetes (10). While it has been traditionally believed that high levels of LDL-C indirectly affect metabolic health by promoting atherosclerosis, new evidence suggests that LDL-C may be directly involved in the pathology of insulin resistance and β-cell dysfunction (11, 12). Notably, recent studies have revealed a correlation between LDL-C subtypes and prediabetes risk. Elevated proportions of small dense LDL (sdLDL) particles, which are more likely to oxidize and penetrate the vascular endothelium, were significantly and positively associated with insulin resistance and prediabetes risk (13). Unfortunately, previous studies on the association between LDL-C and prediabetes have been predominantly cross-sectional, a study design that does not allow for causal relationships between variables. In addition, prior studies lacked subgroup analyses or examination of non-linear associations between these variables. Therefore, this study utilized a publicly accessible database to investigate the exact relationship between LDL-C levels and the risk of prediabetes in a large Chinese population.

## Methods

### Data source

We downloaded data from the Dryad database uploaded by Chen et al. (dataset: https://datadryad.org/stash/dataset/doi:10.5061%2Fdryad.ft8750v). The dataset comprised medical data from 685,277 adults recruited by Rich Healthcare Group for health checkups across 32 districts in 11 Chinese cities (2010–2016). All participants underwent at least two health checkups during this period. According to Dryad’s Terms of Service, researchers are permitted to utilize the data for secondary analysis, exploring new hypotheses, and optimizing data utilization.

### Study population

In the original study, Chen et al. analyzed the association between body mass index (BMI) and diabetes risk. Subjects with the following characteristics were excluded: 1) missing height and weight data; 2) unknown sex; 3) extreme BMI, defined as a BMI <15 or >55 kg/m^2^; 4) missing baseline fasting plasma glucose (FPG) data; 5) diabetes mellitus at baseline; 6) diabetes mellitus status unknown during the follow-up period; and 7) follow-up period of less than 2 years. Ultimately, 211,833 participants were enrolled.

**Figure 1:**
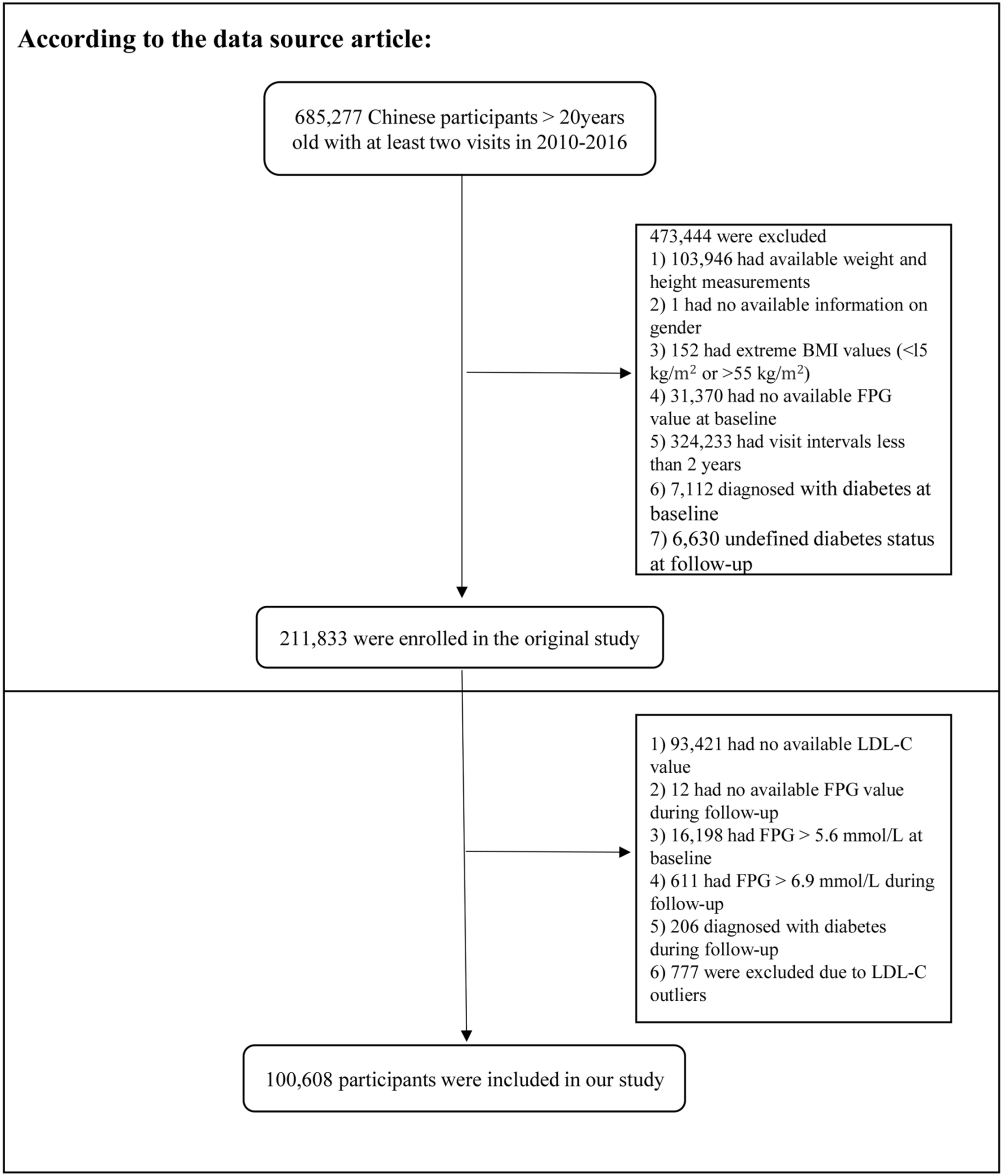
Flowchart for screening research participants.

The study subjects were further included according to the criteria shown in **Figure 1**. The exclusion criteria were as follows: 1) missing LDL-C data at baseline, 2) missing FPG data during follow-up, 3) FPG > 5.6 mmol/L at baseline, 4) FPG > 6.9 mmol/L during follow-up, 5) diagnosis of diabetes mellitus during follow-up, and 6) abnormal LDL-C values (3 standard deviations above or below the mean). Ethical approval for the original study was provided by the Rich Healthcare Group Review Committee. As a secondary analysis, this study did not require separate ethical approval. In addition, the initial study was completed in accordance with the principles of the Declaration of Helsinki. All procedures followed relevant guidelines and regulations.

### Health examinations and laboratory measurements

The researchers used a standardized questionnaire to obtain socio-demographic data on the participants, covering age, gender, lifestyle habits (drinking/smoking status), the presence of chronic diseases (diabetes), and family history (family history of diabetes). Smoking and drinking status were categorized into four categories: never, once, current, and undocumented. Participants’ height, weight, and blood pressure (BP) were measured by a healthcare professional in a standardized environment. Weight was measured with an accuracy of 0.1 kg and height with an accuracy of 0.1 cm.

All participants were required to fast for at least 10 hours prior to the blood biochemistry tests. Venous blood was collected from participants by skilled nursing staff and then analyzed in a standard laboratory using automated analyzers (Beckman Coulter AU5800, Brea, CA, USA) to measure levels of aspartate aminotransferase (AST), high-density lipoprotein cholesterol (HDL-C), alanine aminotransferase (ALT), triglycerides (TG), creatinine (Cr), total cholesterol (TC), blood urea nitrogen (BUN), LDL-C, and FPG.

### Definitions

Prediabetes was defined as the absence of diabetes and FPG levels of 5.6–6.9 mmol/L throughout the follow-up period.

### Missing data processing

In this study, there were 10 variables with missing values. Among them, AST (58,303, 57.95%), smoking status (72,723, 72.28%), and drinking status (72,723, 72.28%) had substantial missingness. AST was first converted to a categorical variable based on tertiles. Individuals with missing data on smoking status, drinking status, and AST were then considered as a separate group (the not-recorded group). In addition, systolic blood pressure (SBP), diastolic blood pressure (DBP), TG, TC, ALT, BUN, and serum creatinine (Scr) were missing in 12, 12, 1, 2, 370, 2,428, and 1,201 cases, respectively. Next, linear regression and 10 iterations were used for the interpolation of missing data for multiple variables.

### Statistical analysis

Continuous variables were presented as means ± standard deviation (SD) if normally distributed, or as median and interquartile range (IQR) if not. Categorical variables were presented as frequencies and percentages. Differences in continuous variables between groups were compared using one-way ANOVA or the Kruskal–Wallis H test, whereas categorical variables were compared using the chi-square test or Fisher’s exact test. The Kaplan–Meier method was used to compare survival and cumulative event rates. The log-rank test was used to analyze the Kaplan–Meier hazard ratio (HR) for adverse events.

We analyzed the association between LDL-C and the risk of prediabetes using multivariate Cox regression. The analysis included unadjusted models, minimally adjusted models (adjusting for gender, age, BMI, SBP, DBP, smoking status, drinking status, and family history of diabetes mellitus), and fully adjusted models (adjusting for gender, age, BMI, SBP, DBP, smoking status, drinking status, family history of diabetes, AST, ALT, HDL-C, TC, TG, BUN, Scr, and baseline FPG). Next, we performed various sensitivity analyses to check the reliability of the findings. We analyzed the raw data without interpolation. To investigate the association between LDL-C and the risk of prediabetes, we excluded individuals aged ≥60 years or with a BMI ≥ 25 kg/m^2^ for further sensitivity analyses. In addition, we used a generalized additive model (GAM) to test the validity of the results. We also calculated E-values to examine the possibility of unmeasured confounding.

A Cox proportional risk regression model combining cubic spline functions and smooth curve fitting was used to explore the non-linear relationship between LDL-C and prediabetes. A two-piecewise Cox proportional risk regression model was constructed using a recursive algorithm to identify the inflection point and analyze data on both sides of the inflection point. The optimal model was determined by a log-likelihood ratio test. Next, the Cox proportional risk model was used to stratify the population into different subgroups. Likelihood ratio tests were used to confirm interactions between the different subgroups. Finally, mediation analyses were performed, and the proportions of the mediating effects of BMI, age, SBP, DBP, smoking status, and drinking status were assessed using the bootstrap method. p-Value < 0.05 was considered statistically significant. All analyses were performed using the R software version 3.6.1 (http://www.r-project.org, R Foundation) and EmpowerStats^®^ (version 6.0, www.empowerstats.com, X&Y Solutions, Inc., Boston, MA, USA).

## Results

### Characteristics of participants

A total of 100,608 individuals with prediabetes at baseline were included in this study. Of these individuals, 52.11% were male, and the mean age was 42.7 ± 12.51 years. After a mean follow-up of 3.12 years, 12,433 individuals were finally diagnosed with prediabetes. Participants were categorized into four groups based on quartiles of LDL-C (Q1 ≤ 2.26, 2.26 < Q2 ≤ 2.66, 2.66 < Q3 ≤ 3.1, and Q4 > 3.1). Age, BMI, fasting blood glucose (FBG), TC, TG, ALT, and AST showed a significant increase with increasing LDL-C (all p-values < 0.001). The baseline characteristics of all participants are presented in **Table 1**.

**Table 1:** The baseline characteristics of participants.

LDL-C (mmol/L)	Q1 (≤2.26)	Q2 (2.26 to ≤ 2.66)	Q3 (2.66 to ≤ 3.1)	Q4 (>3.1)	p-Value
Participants	25,087	25,208	24,807	25,506	
Age (year)	39.36 ± 11.14	41.62 ± 11.86	43.65 ± 12.44	47.22 ± 13.14	<0.001
Height (cm)	166.08 ± 8.13	166.30 ± 8.22	166.45 ± 8.32	166.10 ± 8.49	<0.001
Weight (kg)	61.84 ± 11.41	63.60 ± 11.72	64.99 ± 12.03	66.04 ± 12.01	<0.001
BMI (kg/m^2^)	22.32 ± 3.12	22.89 ± 3.18	23.34 ± 3.21	23.82 ± 3.17	<0.001
SBP (mmHg)	115.20 ± 14.94	116.96 ± 15.69	118.70 ± 16.18	121.31 ± 16.76	<0.001
DBP (mmHg)	71.79 ± 10.32	73.06 ± 10.60	74.25 ± 10.72	75.84 ± 10.95	<0.001
FPG (mmol/L)	4.73 ± 0.49	4.77 ± 0.47	4.80 ± 0.48	4.84 ± 0.47	<0.001
TC (mmol/L)	3.83 ± 0.48	4.42 ± 0.39	4.90 ± 0.40	5.73 ± 0.60	<0.001
TG (mmol/L)	1.11 ± 1.04	1.20 ± 0.86	1.33 ± 0.85	1.55 ± 0.94	<0.001
HDL-C (mmol/L)	1.32 ± 0.29	1.38 ± 0.29	1.39 ± 0.31	1.41 ± 0.33	<0.001
ALT (U/L)	20.50 ± 22.22	21.85 ± 22.10	23.53 ± 20.04	26.18 ± 20.48	<0.001
BUN (mmol/L)	4.45 ± 1.15	4.58 ± 1.14	4.68 ± 1.15	4.81 ± 1.17	<0.001
Scr (μmol/L)	68.19 ± 15.96	69.58 ± 16.33	70.59 ± 15.46	71.28 ± 15.94	<0.001
Gender					<0.001
Male	12,043 (48.00%)	12,955 (51.39%)	13,437 (54.17%)	13,994 (54.87%)	
Female	13,044 (52.00%)	12,253 (48.61%)	11,370 (45.83%)	11,512 (45.13%)	
AST					<0.001
Low	4,348 (17.33%)	3,873 (15.36%)	3,334 (13.44%)	2,587 (10.14%)	
Moderate	3,405 (13.57%)	3,601 (14.29%)	3,632 (14.64%)	3,572 (14.00%)	
High	2,777 (11.07%)	3,198 (12.69%)	3,601 (14.52%)	4,377 (17.16%)	
Not recorded	14,557 (58.03%)	14,536 (57.66%)	14,240 (57.40%)	14,970 (58.69%)	
Prediabetes					<0.001
No	22,585 (90.03%)	22,250 (88.27%)	21,623 (87.16%)	21,717 (85.14%)	
Yes	2,502 (9.97%)	2,958 (11.73%)	3,184 (12.84%)	3,789 (14.86%)	
Smoking status					<0.001
Current smoker	1,046 (4.17%)	1,312 (5.20%)	1,387 (5.59%)	1,660 (6.51%)	
Ex-smoker	272 (1.08%)	282 (1.12%)	260 (1.05%)	284 (1.11%)	
Never-smoker	6,054 (24.13%)	5,286 (20.97%)	4,958 (19.99%)	5,084 (19.93%)	
Not recorded	17,715 (70.61%)	18,328 (72.71%)	18,202 (73.37%)	18,478 (72.45%)	
Drinking status					<0.001
Current drinker	140 (0.56%)	167 (0.66%)	167 (0.67%)	185 (0.73%)	
Ex-drinker	1,143 (4.56%)	1,164 (4.62%)	1,111 (4.48%)	1,164 (4.56%)	
Never-drinker	6,089 (24.27%)	5,549 (22.01%)	5,327 (21.47%)	5,679 (22.27%)	
Not recorded	17,715 (70.61%)	18,328 (72.71%)	18,202 (73.37%)	18,478 (72.45%)	
Family history of diabetes					0.168
No	24,538 (97.81%)	24,627 (97.70%)	24,302 (97.96%)	24,926 (97.73%)	
Yes	549 (2.19%)	581 (2.30%)	505 (2.04%)	580 (2.27%)	

LDL-C, low-density lipoprotein cholesterol; BMI, body mass index; FPG, fasting plasma glucose; TC, total cholesterol; TG, triglycerides; HDL-C, high-density lipoprotein cholesterol; ALT, alanine aminotransferase; BUN, blood urea nitrogen; AST, aspartate aminotransferase.

### The incidence rate of prediabetes

**Figure 2:**
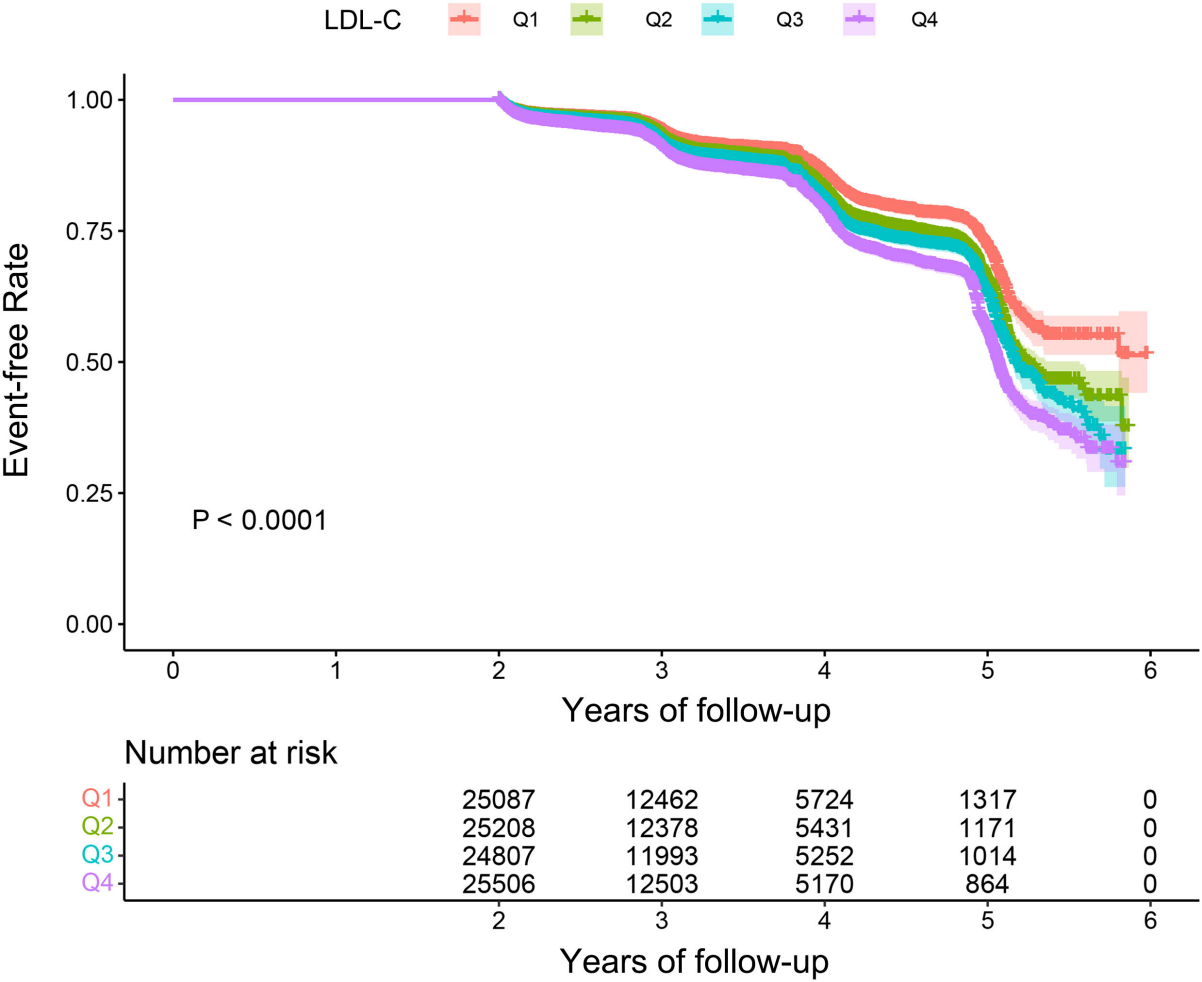
Kaplan–Meier event-free survival curve. Kaplan–Meier analysis of incident prediabetes based on LDL-C quartiles (log-rank, p < 0.0001). LDL-C, low-density lipoprotein cholesterol.

The overall incidence rate of prediabetes among the 100,608 participants during the follow-up period was 12.36%. The incidence rates for the four LDL-C groups were as follows: Q1, 9.97%; Q2, 11.73%; Q3, 12.84%; and Q4, 14.86% (p < 0.001) (**Figure 2**). In addition, the cumulative incidence rate in the total population was 3,583.5/100,000 person-years. In the four LDL-C groups, 2,941.25/100,000 person-years, 3,265.78/100,000 person-years, 3,696.47/100,000 person-years, and 4,409.23/100,000 person-years were observed. The prevalence and cumulative incidence of prediabetes were higher in participants with higher LDL-C compared to those with lower LDL-C (p < 0.001) (**Table 2**).

**Table 2:** Incidence rate of prediabetes.

LDL-C	Participants (n)	Prediabetes events (n)	Cumulative incidence (%)	Per 100,000 person-year
Total	100,608	12,433	12.36	3,583.5
Q1	25,087	2,502	9.97	2,941.25
Q2	25,208	2,958	11.73	3,265.78
Q3	24,807	3,184	12.84	3,696.47
Q4	25,506	3,789	14.86	4,409.23
p for trend			<0.001	<0.001

LDL-C, low-density lipoprotein cholesterol.

### The relationship between LDL-C and prediabetes

In this study, we used three different Cox proportional risk regression models to assess the relationship between LDL-C and prediabetes (**Table 3**). In the non-adjusted model, the risk of prediabetes increased by 31% for every 1-unit increase in LDL-C, with an HR of 1.31 (95% CI: 1.27–1.35; p < 0.0001). In minimally adjusted models adjusting for sex, age, SBP, DBP, family history of diabetes, drinking status, smoking status, and BMI, the HR (95% CI) was 1.06 (1.03, 1.10). The fully adjusted model was further adjusted for biochemical markers (AST, ALT, HDL-C, TC, TG, BUN, Scr, and baseline FPG), and the association between LDL-C and prediabetes remained statistically significant (HR: 1.49, 95% CI: 1.40–1.58; p < 0.0001). The study data showed a 49% increase in the incidence of prediabetes for each unit increase in LDL-C. Furthermore, we converted LDL-C to quartiles, and the correlation between LDL-C and prediabetes remained significant. In the fully adjusted model, the prevalence of prediabetes in the fourth quartile of LDL-C was 50% higher than the prevalence of prediabetes in the first quartile, and a test for trend found that the risk of prediabetes increased significantly with increasing LDL-C (p < 0.001).

**Table 3:** Relationship between LDL-C and incident prediabetes in different models.

Exposure	Non-adjusted model (HR, 95% CI, p)	Mini-adjusted mode (HR, 95% CI, p)	Fully adjusted mode (HR, 95% CI, p)	GAM (HR, 95% CI, p)
LDL-C	1.31 (1.27, 1.35) <0.0001	1.06 (1.03, 1.10) <0.0001	1.49 (1.40, 1.58) <0.0001	1.55 (1.45, 1.64) <0.0001
LDL-C (quartile)				
Q1	Reference	Reference	Reference	Reference
Q2	1.20 (1.14, 1.27) <0.0001	1.06 (1.01, 1.12) 0.0207	1.15 (1.10, 1.22) <0.0001	1.17 (1.10, 1.23) <0.0001
Q3	1.34 (1.27, 1.41) <0.0001	1.07 (1.02, 1.13) 0.0086	1.25 (1.17, 1.34) <0.0001	1.28 (1.19, 1.37) <0.0001
Q4	1.58 (1.51, 1.67) <0.0001	1.10 (1.04, 1.16) 0.0002	1.48 (1.36, 1.62) <0.0001	1.50 (1.37, 1.64) <0.0001
p for trend	<0.0001	<0.0001	<0.0001	<0.0001

Non-adjusted model: no adjustment for other covariates. Minimally adjusted model: adjusted for gender, age, BMI, SBP, DBP, smoking status, drinking status, and family history of diabetes mellitus. Fully adjusted model: adjusted for gender, age, BMI, SBP, DBP, smoking status, drinking status, family history of diabetes, AST, ALT, HDL-C, TC, TG, BUN, Scr, and baseline FPG. GAM: all covariates listed in **Table 1** were adjusted. Abbreviations: HR, hazard ratio; CI, confidence interval; GAM, generalized additive model; LDL-C, low-density lipoprotein cholesterol; BMI, body mass index; AST, aspartate aminotransferase; ALT, alanine aminotransferase; HDL-C, high-density lipoprotein cholesterol; TC, total cholesterol; TG, triglycerides; BUN, blood urea nitrogen; FPG, fasting plasma glucose.

### Sensitivity analysis

First, we fitted the data by the smoothing function of the GAM, which was consistent with the results of the fully adjusted model (HR: 1.55, 95% CI: 1.45–1.64, p < 0.0001) (**Table 3**). Next, we performed additional analyses of the data without multiple interpolations. After fully adjusting for confounding covariates, LDL-C remained significantly and positively associated with the risk of prediabetes (HR: 1.43, 95% CI: 1.31–1.55; p < 0.0001) (**Table 4**, Model 1). In addition, we analyzed individuals with BMI < 25 kg/m^2^. After adjusting for confounding covariates, there was also a positive association between LDL-C and risk of prediabetes, yielding an HR of 1.43 (95% CI: 1.31–1.55, p < 0.0001) (**Table 4**, Model 2). After excluding individuals over 60 years of age, the analysis showed that LDL-C remained positively associated with the risk of prediabetes (HR: 1.49, 95% CI: 1.39–1.60) (**Table 4**, Model 3). Additionally, we computed an E-value to assess the vulnerability of the study results to potential unobserved confounding factors. The resulting E-value (2.06) showed a higher level of statistical significance compared to the relative risk associated with unmeasured confounders and LDL-C (1.55), suggesting that unrecognized confounders had a negligible effect on the results.

**Table 4:** Relationship between LDL-C and prediabetes in different sensitivity analyses.

Exposure	Model 1	Model 2	Model 3
	(HR, 95% CI, p)	(HR, 95% CI, p)	(HR, 95% CI, p)
LDL-C	1.43 (1.31, 1.55) <0.0001	1.43 (1.31, 1.55) <0.0001	1.49 (1.39, 1.60) <0.0001
LDL-C (quartile)			
Q1	Reference	Reference	Reference
Q2	1.13 (1.08, 1.19) <0.0001	1.11 (1.03, 1.20) 0.0053	1.16 (1.08, 1.23) <0.0001
Q3	1.29 (1.20, 1.38) <0.0001	1.23 (1.13, 1.34) <0.0001	1.26 (1.17, 1.36) <0.0001
Q4	1.36 (1.23, 1.48) <0.0001	1.42 (1.26, 1.60) <0.0001	1.52 (1.38, 1.68) <0.0001
p for trend	<0.0001	<0.0001	<0.0001

Model 1 was a sensitivity analysis of the raw data before interpolation. We adjusted for gender, age, BMI, SBP, DBP, smoking status, drinking status, family history of diabetes, AST, ALT, HDL-C, TC, TG, BUN, Scr, and baseline FPG. Model 2 was a sensitivity analysis of participants with BMI < 25 kg/m^2^. We adjusted for gender, age, BMI, SBP, DBP, smoking status, drinking status, family history of diabetes, AST, ALT, HDL-C, TC, TG, BUN, Scr, and baseline FPG. Model 3 was a sensitivity analysis of participants aged <60 years. We adjusted for gender, age, BMI, SBP, DBP, smoking status, drinking status, family history of diabetes, AST, ALT, HDL-C, TC, TG, BUN, Scr, and baseline FPG.

HR, hazard ratio; CI, confidence interval; LDL-C, low-density lipoprotein cholesterol; BMI, body mass index; AST, aspartate aminotransferase; ALT, alanine aminotransferase; HDL-C, high-density lipoprotein cholesterol; TC, total cholesterol; TG, triglycerides; BUN, blood urea nitrogen; FPG, fasting plasma glucose.

### Non-linear association between LDL-C and prediabetes

**Figure 3:**
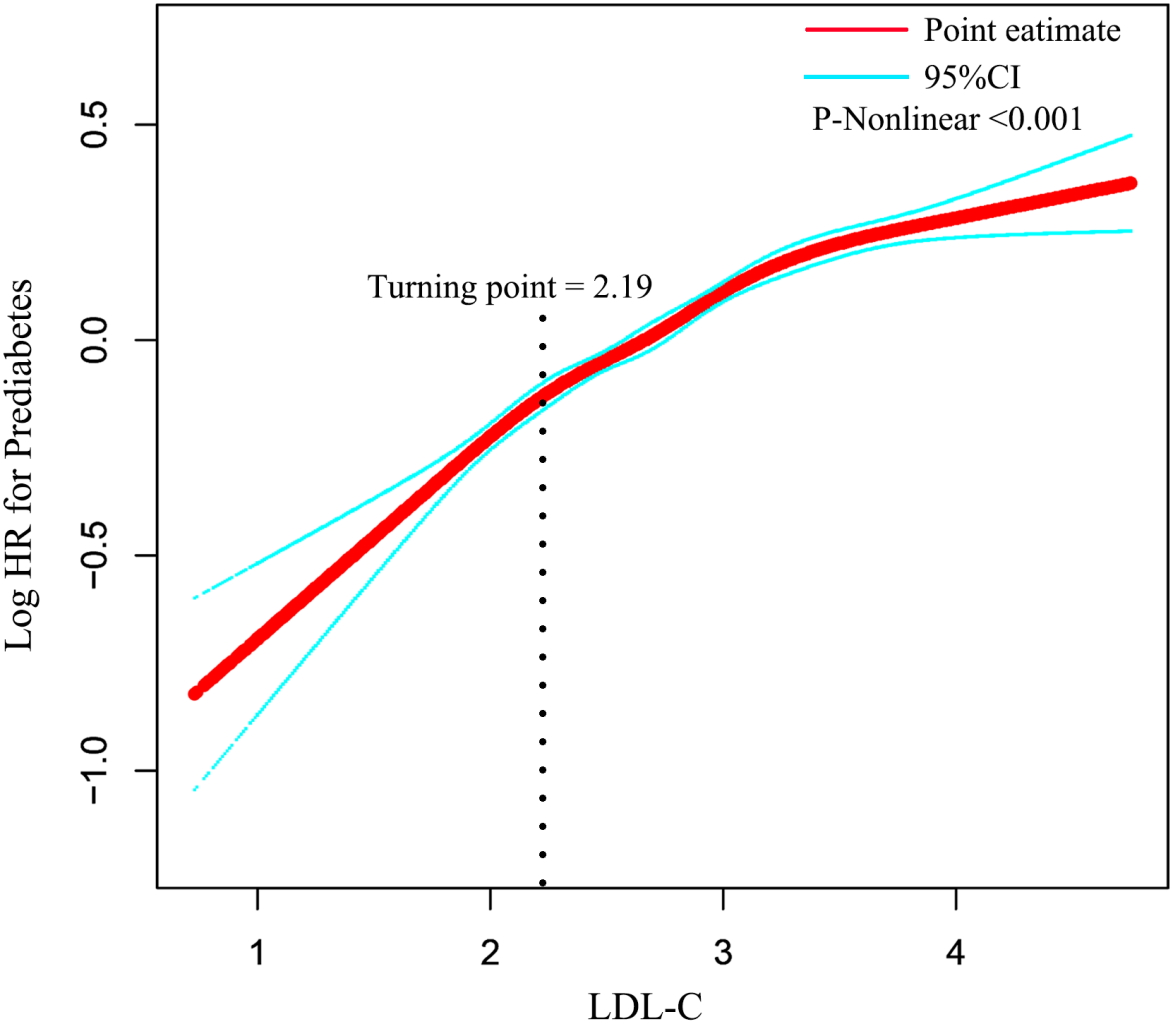
The non-linear relationship between LDL-C and incident prediabetes. Adjusted for gender, age, BMI, SBP, DBP, smoking status, drinking status, family history of diabetes, AST, ALT, HDL-C, TC, TG, BUN, Scr, and baseline FPG. HR, hazard ratio; CI, confidence interval; LDL-C, low-density lipoprotein cholesterol; BMI, body mass index; AST, aspartate aminotransferase; ALT, alanine aminotransferase; HDL-C, high-density lipoprotein cholesterol; TC, total cholesterol; TG, triglycerides; BUN, blood urea nitrogen; FPG, fasting plasma glucose.

We found a non-linear association between LDL-C and prediabetes by combining cubic spline function and smooth curve fitting (**Table 5**). The two-stage Cox proportional risk regression model found an inflection point of 2.19 for LDL-C (p < 0.001 for the log-likelihood ratio test). When LDL-C ≤ 2.19, LDL-C was positively associated with the risk of prediabetes (HR: 2.02, 95% CI: 1.73–2.36, p < 0.0001). In contrast, when LDL-C > 2.19, LDL-C was associated with a reduced relative risk of prediabetes (HR: 1.49, 95% CI: 1.39–1.59, p < 0.0001) (**Figure 3**).

**Table 5:** The result of the two-piecewise Cox proportional hazards regression model.

Incident prediabetes	HR (95% CI)	p-Value
Fitting by standard Cox proportional hazards regression	1.55 (1.45, 1.65)	<0.0001
Fitting by two-piecewise Cox proportional hazards regression		
Inflection points of LDL-C	2.19	
≤2.19	2.02 (1.73, 2.36)	<0.0001
>2.19	1.49 (1.39, 1.59)	<0.0001
p for log-likelihood ratio test		<0.001

We adjusted for gender, age, BMI, SBP, DBP, smoking status, drinking status, family history of diabetes, AST, ALT, HDL-C, TC, TG, BUN, Scr, and baseline FPG.

HR, hazard ratio; CI, confidence interval; BMI, body mass index; AST, aspartate aminotransferase; ALT, alanine aminotransferase; HDL-C, high-density lipoprotein cholesterol; TC, total cholesterol; TG, triglycerides; BUN, blood urea nitrogen; FPG, fasting plasma glucose.

### Subgroup analysis

**Figure 4:**
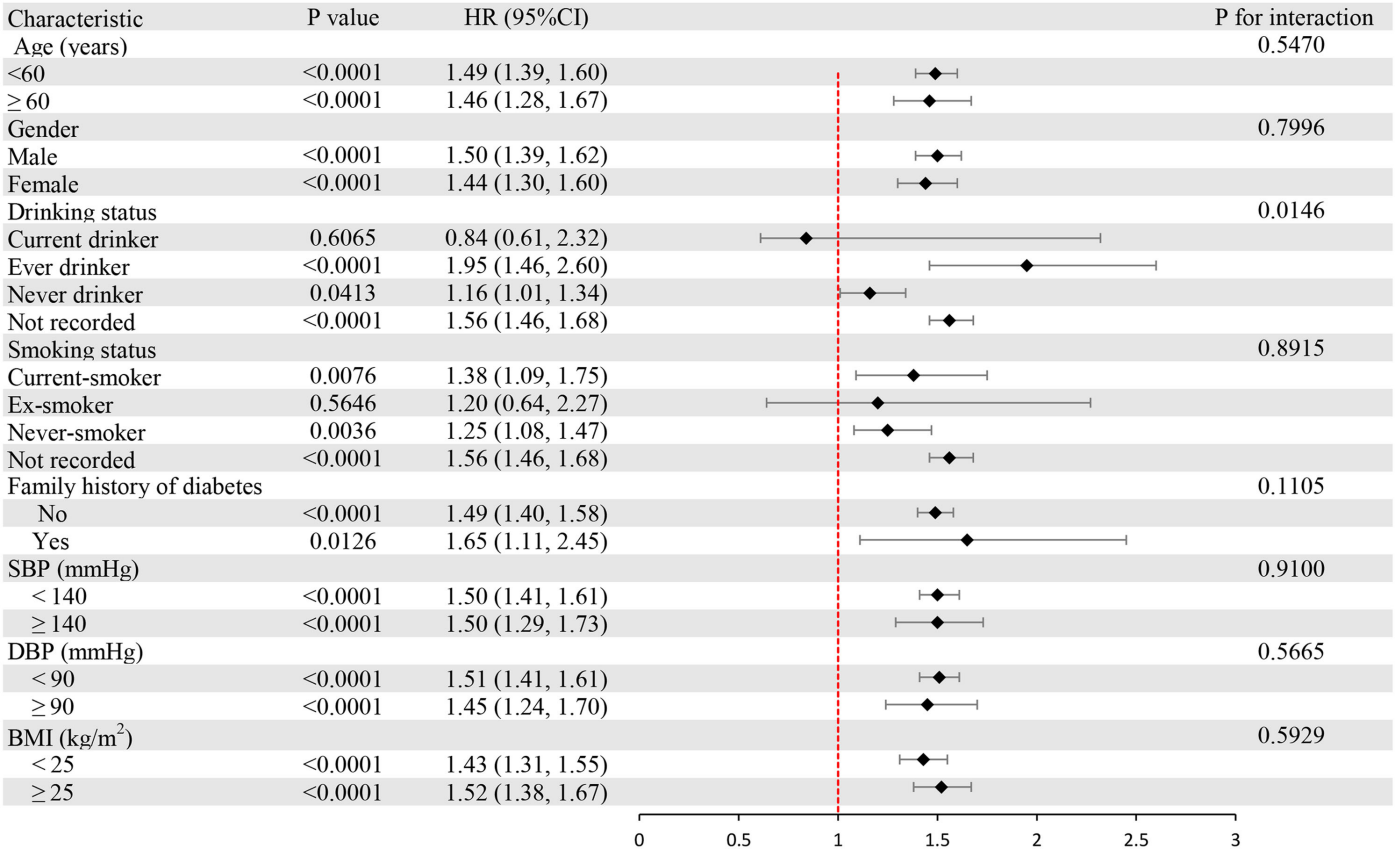
Subgroup analysis of the associations between LDL-C and prediabetes. LDL-C, low-density lipoprotein cholesterol.

We performed subgroup analyses to examine the differences between LDL-C disease and prediabetes in different populations (**Figure 4**). Stratification factors included age, gender, BMI, SBP, DBP, smoking status, drinking status, and family history of diabetes. The results of the study showed a stronger correlation between LDL-C and the risk of prediabetes in individuals under 60 years of age, women, and individuals with body mass index ≥ 25 kg/m^2^ and a family history of diabetes. The interaction results suggest that the positive association between LDL-C and the risk of prediabetes is stable and consistent in the general population.

### Mediation analysis

**Figure 5:**
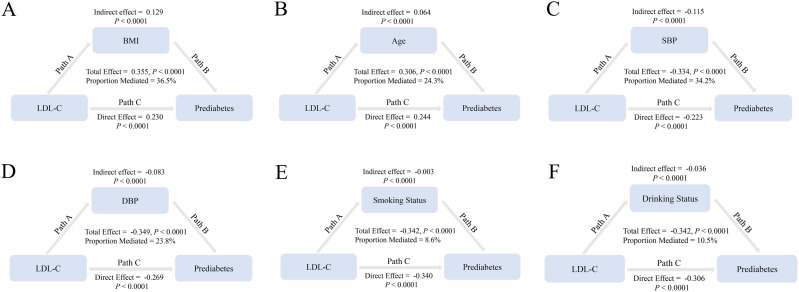
BMI, age, SBP, DBP, smoking status, and drinking status mediate the association of LDL-C with prediabetes. Note: In mediation analyses, adjustments were made for gender, family history of diabetes, AST, ALT, HDL-C, TC, TG, BUN, Scr, and baseline FPG. HR, hazard ratio; CI, confidence interval; BMI, body mass index; LDL-C, low-density lipoprotein cholesterol; AST, aspartate aminotransferase; ALT, alanine aminotransferase; HDL-C, high-density lipoprotein cholesterol; TC, total cholesterol; TG, triglycerides; BUN, blood urea nitrogen; FPG, fasting plasma glucose.

The potential mediating effects of BMI, age, SBP, DBP, smoking status, and drinking status were assessed separately by mediation analysis. **Figure 5** shows that the above variables exerted partial mediating effects on the relationship between LDL-C and prediabetes risk (BMI, 36.5%; age, 24.3%; SBP, 34.2%; DBP, 23.8%; smoking status, 8.6%; and drinking status, 10.5%).

## Discussion

In this study of Chinese adults, we observed a significant positive relationship between LDL-C levels and prediabetes risk. Notably, this association persisted as statistically significant even after adjusting for potential confounding variables. Specifically, for each 1-unit increase in LDL-C, the risk of prediabetes increased by 49%. Participants in the highest LDL-C quartile had 48% higher odds of developing prediabetes compared to those in the lowest quartile. Furthermore, Restricted Cubic Spline (RCS) analysis revealed a non-linear relationship between LDL-C and prediabetes risk, with an inflection point identified at 2.19. Subgroup analyses confirmed that the positive association between LDL-C and prediabetes risk was robust and consistent across the general population.

The current study demonstrates the key role of the interaction between lipid metabolism disorders and glucose metabolism abnormalities in prediabetes (14). Dyslipidemia promotes inflammation and endoplasmic reticulum stress. In addition, lipotoxicity can lead to insulin resistance (IR), playing a critical role in diabetes progression (15). A cross-sectional study including 2,293 adults found that higher TG was significantly associated with an increased risk of prediabetes (OR: 1.96, p < 0.001) (16). Elevated TG levels increase free fatty acids, altering insulin signaling in pancreatic α-cells and promoting excessive glucagon secretion (17). Guo et al. reported that the TG/HDL-C ratio was significantly associated with prediabetes after adjusting for confounders such as age, sex, blood pressure, and FPG (OR: 3.45, 95% CI: 2.42–4.92, p < 0.001) (18), an association also observed in non-obese individuals with normal LDL-C (19). LDL-C is a primary target for lipid-lowering therapy and a major parameter for cardiovascular risk assessment (20). Currently, the influence of LDL-C on the development of diabetes remains controversial. Similar to type 2 diabetes (T2D), the typical pattern of dyslipidemia in prediabetes includes elevated serum triglycerides, decreased HDL-C, and elevated total LDL particles (21). A Thai cross-sectional study found that after adjusting for confounders, individuals with high LDL-C levels were 1.96 times more likely to develop prediabetes than those with normal LDL-C levels (95% CI: 1.30–2.96, p < 0.01) (22). Moreover, there is an association between different LDL-C subtypes and prediabetes. SdLDL particles are defined as LDL with an average diameter of <25.5 nm. Tiny-sized sdLDL particles have a lower affinity for LDL-C receptors and are able to penetrate the arterial wall more readily (23). A prospective study showed that sdLDL-C levels were significantly associated with inflammatory markers (hypersensitive c-reactive protein (hs-CRP) levels, total white blood cell count, and fibrinogen) in a non-diabetic population. After adjusting for confounders, people with higher sdLDL-C levels had a significantly increased risk of prediabetes (13). Currently, some new research evidence suggests that LDL-C may have a protective role in the progression of prediabetes. Reports indicate that individuals with familial hypercholesterolemia characterized by high LDL-C levels have a lower prevalence of diabetes than unaffected relatives (24). The American Heart Association (AHA) also suggests that the long-term use of tamsulosin may increase the risk of new-onset diabetes, an effect that may be attributable to the indirect effects of the drug itself on β-cell function rather than to the direct consequences of lower LDL-C (25). In this cohort study, we delved into the relationship between LDL-C levels and prediabetes in a Chinese adult population. To ensure the robustness of the findings, we controlled for more potential confounders such as Scr, AST, ALT, and family history of diabetes. In addition, a series of sensitivity analyses (target independent variable transformations, subgroup analyses, and supplemental analyses using GAM) demonstrated the robustness of the findings. This study informs the dynamic monitoring of LDL-C levels to identify those at high risk of prediabetes. More aggressive countermeasures (e.g., dietary modification and regular aerobic exercise) at an early stage can significantly reduce the potential risk.

We propose potential mechanisms linking LDL-C to prediabetes. First, elevated LDL-C is often accompanied by metabolic abnormalities like visceral fat accumulation and hypertriglyceridemia, creating a vicious cycle of insulin resistance (26). Lipid deposition in the liver and muscle interferes with insulin signaling and reduces glucose uptake (27). Second, high levels of LDL-C [especially oxidized LDL (ox-LDL)] can interfere with insulin signaling pathways by inducing oxidative stress and chronic inflammation (28, 29). Ox-LDL activates scavenger receptors on the surface of macrophages, promotes the release of inflammatory factors (e.g., TNF-α and IL-6), and inhibits the phosphorylation of insulin receptor substrate-1 (IRS-1), thereby exacerbating IR (30). Clinical studies have shown that ox-LDL levels are significantly elevated in prediabetic patients and strongly positively correlate with glycated hemoglobin (HbA1c), suggesting that ox-LDL may accelerate glucose metabolism disorders by impairing microvascular endothelial function (31). Finally, excess LDL-C accumulates in pancreatic β-cells, triggering lipotoxic effects. Free cholesterol accumulation leads to increased mitochondrial membrane permeability, increased reactive oxygen species (ROS) generation, and the inhibition of insulin secretion (32, 33). These potential mechanisms provide a pathophysiologic explanation for the association between LDL-C and the development of prediabetes.

Our study has several strengths. First, the large sample size (n = 100,608) and extended follow-up (mean 3.12 years) from a Chinese cohort enhanced result reliability and generalizability within this population. Second, we addressed the issue of missing data using multiple interpolations, which maximizes statistical power and reduces the potential bias caused by missing covariates. Third, we conducted multiple sensitivity analyses to ensure the robustness of our results. We performed additional analyses of the data before interpolation. We analyzed the association between LDL-C and prediabetes again after excluding individuals with a BMI ≥ 25 kg/m^2^ or an age ≥ 60 years. In addition, we used a generalized summation model to fit the data to further validate the findings. Fourth, we calculated E-values to explore the possibility of unmeasured confounding. However, there are some limitations to our study. First, the participants in this study were of East Asian origin. There may be potential differences across racial groups. East Asian populations have a higher frequency of PCSK9 gene variants, which may influence the strength of the association between LDL-C metabolism and glucose metabolism (34). Furthermore, a Western diet high in saturated fats may amplify the metabolic toxicity of LDL-C. The core mechanisms linking LDL-C to prediabetes risk may be universal, but risk thresholds require calibration based on racial specificity. Second, similar to all observational studies, despite controlling for known potential confounders, the presence of uncontrolled or unmeasured confounders, including diet and exercise, cannot be completely excluded. In addition, in the original study, LDL-C levels were measured only at baseline. Future studies should monitor changes in LDL-C during follow-up to examine the association of dynamic changes in LDL-C with prediabetes.

## Conclusion

This study confirms a non-linear positive association between LDL-C levels and prediabetes risk in Chinese adults. The dynamic monitoring of LDL-C may help identify individuals at high risk for prediabetes. Timely dietary and lifestyle interventions could help reduce the risk. These findings provide new insights into the prevention and treatment of prediabetes. Further research is needed to elucidate the underlying mechanisms.

## Data Availability

The original contributions presented in the study are included in the article/supplementary material. Further inquiries can be directed to the corresponding author.

## References

[1] Duan D Kengne AP Echouffo-Tcheugui JB. Screening for diabetes and prediabetes. Endocrinol Metab Clin North Am. (2021) 50:369–85. doi: 10.1016/j.ecl.2021.05.002 PMC8375583 34399951

[2] Echouffo-Tcheugui JB Perreault L Ji L Dagogo-Jack S. Diagnosis and management of prediabetes: A review. JAMA. (2023) 329:1206–16. doi: 10.1001/jama.2023.4063 37039787

[3] Saeedi P Petersohn I Salpea P Malanda B Karuranga S Unwin N. Global and regional diabetes prevalence estimates for 2019 and projections for 2030 and 2045: Results from the International Diabetes Federation Diabetes Atlas, 9(th) edition. Diabetes Res Clin Pract. (2019) 157:107843. doi: 10.1016/j.diabres.2019.107843 31518657

[4] Tabak AG Herder C Rathmann W Brunner EJ Kivimaki M. Prediabetes: a high-risk state for diabetes development. Lancet. (2012) 379:2279–90. doi: 10.1016/S0140-6736(12)60283-9 PMC3891203 22683128

[5] Brannick B Dagogo-Jack S. Prediabetes and cardiovascular disease: pathophysiology and interventions for prevention and risk reduction. Endocrinol Metab Clin North Am. (2018) 47:33–50. doi: 10.1016/j.ecl.2017.10.001 29407055 PMC5806140

[6] Beals JW Kayser BD Smith GI Schweitzer GG Kirbach K Kearney ML. Dietary weight loss-induced improvements in metabolic function are enhanced by exercise in people with obesity and prediabetes. Nat Metab. (2023) 5:1221–35. doi: 10.1038/s42255-023-00829-4 PMC10515726 37365374

[7] Knowler WC Barrett-Connor E Fowler SE Hamman RF Lachin JM Walker EA. Reduction in the incidence of type 2 diabetes with lifestyle intervention or metformin. N Engl J Med. (2002) 346:393–403. doi: 10.1056/NEJMoa012512 11832527 PMC1370926

[8] Jiang ZZ Zhu JB Shen HL Zhao SS Tang YY Tang SQ. A high triglyceride-glucose index value is associated with an increased risk of carotid plaque burden in subjects with prediabetes and new-onset type 2 diabetes: A real-world study. Front Cardiovasc Med. (2022) 9:832491. doi: 10.3389/fcvm.2022.832491 35310963 PMC8927542

[9] Zheng X Zhang X Han Y Hu H Cao C. Nonlinear relationship between atherogenic index of plasma and the risk of prediabetes: a retrospective study based on Chinese adults. Cardiovasc Diabetol. (2023) 22:205. doi: 10.1186/s12933-023-01934-0 37563588 PMC10416492

[10] Jin JL Zhang HW Cao YX Liu HH Hua Q Li YF. Long-term prognostic utility of low-density lipoprotein (LDL) triglyceride in real-world patients with coronary artery disease and diabetes or prediabetes. Cardiovasc Diabetol. (2020) 19:152. doi: 10.1186/s12933-020-01125-1 32981521 PMC7520976

[11] Bjornstad P Eckel RH. Pathogenesis of lipid disorders in insulin resistance: a brief review. Curr Diabetes Rep. (2018) 18:127. doi: 10.1007/s11892-018-1101-6 PMC6428207 30328521

[12] Kruit JK Brunham LR Verchere CB Hayden MR. HDL and LDL cholesterol significantly influence beta-cell function in type 2 diabetes mellitus. Curr Opin Lipidol. (2010) 21:178–85. doi: 10.1097/MOL.0b013e328339387b 20463468

[13] Hsu SH Jang MH Torng PL Su TC. Positive association between small dense low-density lipoprotein cholesterol concentration and biomarkers of inflammation, thrombosis, and prediabetes in non-diabetic adults. J Atheroscler Thromb. (2019) 26:624–35. doi: 10.5551/jat.43968 PMC6629751 30587667

[14] Kane JP Pullinger CR Goldfine ID Malloy MJ. Dyslipidemia and diabetes mellitus: Role of lipoprotein species and interrelated pathways of lipid metabolism in diabetes mellitus. Curr Opin Pharmacol. (2021) 61:21–7. doi: 10.1016/j.coph.2021.08.013 34562838

[15] Glass CK Olefsky JM. Inflammation and lipid signaling in the etiology of insulin resistance. Cell Metab. (2012) 15:635–45. doi: 10.1016/j.cmet.2012.04.001 PMC4156155 22560216

[16] Bhowmik B Siddiquee T Mujumder A Afsana F Ahmed T Mdala IA. Serum lipid profile and its association with diabetes and prediabetes in a rural Bangladeshi population. Int J Environ Res Public Health. (2018) 15:1944. doi: 10.3390/ijerph15091944 30200612 PMC6165005

[17] Manell H Kristinsson H Kullberg J Ubhayasekera SJK Morwald K Staaf J. Hyperglucagonemia in youth is associated with high plasma free fatty acids, visceral adiposity, and impaired glucose tolerance. Pediatr Diabetes. (2019) 20:880–91. doi: 10.1111/pedi.12890 31271247

[18] Guo W Qin P Lu J Li X Zhu W Xu N. Diagnostic values and appropriate cutoff points of lipid ratios in patients with abnormal glucose tolerance status: a cross-sectional study. Lipids Health Dis. (2019) 18:130. doi: 10.1186/s12944-019-1070-z 31153374 PMC6545201

[19] Wu L Wu X Hu H Wan Q. Association between triglyceride-to-high-density lipoprotein cholesterol ratio and prediabetes: a cross-sectional study in Chinese non-obese people with a normal range of low-density lipoprotein cholesterol. J Transl Med. (2022) 20:484. doi: 10.1186/s12967-022-03684-1 36273126 PMC9588227

[20] Neves JS Newman C Bostrom JA Buysschaert M Newman JD Medina JL. Management of dyslipidemia and atherosclerotic cardiovascular risk in prediabetes. Diabetes Res Clin Pract. (2022) 190:109980. doi: 10.1016/j.diabres.2022.109980 35787415

[21] Verges B. Pathophysiology of diabetic dyslipidaemia: where are we? Diabetologia. (2015) 58:886–99. doi: 10.1007/s00125-015-3525-8 PMC4392164 25725623

[22] Apidechkul T Chomchiei C Upala P Tamornpark R. Epidemiology of prediabetes mellitus among hill tribe adults in Thailand. PloS One. (2022) 17:e0271900. doi: 10.1371/journal.pone.0271900 35877774 PMC9312415

[23] Ivanova EA Myasoedova VA Melnichenko AA Grechko AV Orekhov AN. Small dense low-density lipoprotein as biomarker for atherosclerotic diseases. Oxid Med Cell Longev. (2017) 2017:1273042. doi: 10.1155/2017/1273042 28572872 PMC5441126

[24] Besseling J Kastelein JJ Defesche JC Hutten BA Hovingh GK. Association between familial hypercholesterolemia and prevalence of type 2 diabetes mellitus. JAMA. (2015) 313:1029–36. doi: 10.1001/jama.2015.1206 25756439

[25] Newman CB Preiss D Tobert JA Jacobson TA Page RL 2nd Goldstein LB. Statin safety and associated adverse events: A scientific statement from the american heart association. Arterioscler Thromb Vasc Biol. (2019) 39:e38–81. doi: 10.1161/ATV.0000000000000073 30580575

[26] Li B Liu Y Zhou X Chen L Yan L Tang X. Remnant cholesterol is more positively related to diabetes, prediabetes, and insulin resistance than conventional lipid parameters and lipid ratios: A multicenter, large sample survey. J Diabetes. (2024) 16:e13592. doi: 10.1111/1753-0407.13592 39136535 PMC11320755

[27] Corcoran MP Lamon-Fava S Fielding RA. Skeletal muscle lipid deposition and insulin resistance: effect of dietary fatty acids and exercise. Am J Clin Nutr. (2007) 85:662–77. doi: 10.1093/ajcn/85.3.662 17344486

[28] Jiang W Gan C Zhou X Yang Q Chen D Xiao H. Klotho inhibits renal ox-LDL deposition via IGF-1R/RAC1/OLR1 signaling to ameliorate podocyte injury in diabetic kidney disease. Cardiovasc Diabetol. (2023) 22:293. doi: 10.1186/s12933-023-02025-w 37891556 PMC10612302

[29] Scazzocchio B Vari R D’Archivio M Santangelo C Filesi C Giovannini C. Oxidized LDL impair adipocyte response to insulin by activating serine/threonine kinases. J Lipid Res. (2009) 50:832–45. doi: 10.1194/jlr.M800402-JLR200 PMC2666169 19136667

[30] Jiang G Li J Niu S Dong R Chen Y Bi W. LY86 facilitates ox-LDL-induced lipid accumulation in macrophages by upregulating SREBP2/HMGCR expression. BMC Cardiovasc Disord. (2024) 24:289. doi: 10.1186/s12872-024-03957-1 38822281 PMC11140969

[31] Hussein OA Gefen Y Zidan JM Karochero EY Luder AS Assy NN. LDL oxidation is associated with increased blood hemoglobin A1c levels in diabetic patients. Clin Chim Acta. (2007) 377:114–8. doi: 10.1016/j.cca.2006.09.002 17070510

[32] Perego C Da Dalt L Pirillo A Galli A Catapano AL Norata GD. Cholesterol metabolism, pancreatic beta-cell function and diabetes. Biochim Biophys Acta Mol Basis Dis. (2019) 1865:2149–56. doi: 10.1016/j.bbadis.2019.04.012 31029825

[33] Guevara-Olaya L Chimal-Vega B Castaneda-Sanchez CY Lopez-Cossio LY Pulido-Capiz A Galindo-Hernandez O. LDL promotes disorders in beta-cell cholesterol metabolism, implications on insulin cellular communication mediated by EVs. Metabolites. (2022) 12:754. doi: 10.3390/metabo12080754 36005626 PMC9415214

[34] Stoekenbroek RM Lambert G Cariou B Hovingh GK. Inhibiting PCSK9 - biology beyond LDL control. Nat Rev Endocrinol. (2018) 15:52–62. doi: 10.1038/s41574-018-0110-5 30367179

